# Spatiotemporal quantification of subcellular ATP levels in a single HeLa cell during changes in morphology

**DOI:** 10.1038/srep16874

**Published:** 2015-11-17

**Authors:** Rika Suzuki, Kohji Hotta, Kotaro Oka

**Affiliations:** 1 Center for Biosciences and Informatics, School of Fundamental Sciences and Technology, Keio University, 3-14-1 Hiyoshi, Kohoku-ku, Yokohama, Kanagawa, Japan

## Abstract

The quantitative relationship between change in cell shape and ATP consumption is an unsolved problem in cell biology. In this study, a simultaneous imaging and image processing analysis allowed us to observe and quantify these relationships under physiological conditions, for the first time. We focused on two marginal regions of cells: the microtubule-rich ‘lamella’ and the actin-rich ‘peripheral structure’. Simultaneous imaging and correlation analysis revealed that microtubule dynamics cause lamellar shape change accompanying an increase in ATP level. Also, image processing and spatiotemporal quantification enabled to visualize a chronological change of the relationships between the protrusion length and ATP levels, and it suggested they are influencing each other. Furthermore, inhibition of microtubule dynamics diminished motility in the peripheral structure and the range of fluctuation of ATP level in the lamella. This work clearly demonstrates that cellular motility and morphology are regulated by ATP-related cooperative function between microtubule and actin dynamics.

Adenosine triphosphate (ATP) is a major energy source for cells, and is used in muscle contraction^1^, neuronal activity^2^, organ development^3^, and many other physiological phenomena. Investigations into intracellular ATP levels have been limited, mostly centered on how they change in responses to 2-deoxyglucose (2-DG) or glucose, which perturb energy metabolism^4^,^5^,^6^, and during hypoxia or excitotoxicity^7^,^8^,^9^. The nature of ATP fluctuation in living cells under normal and physiological conditions is still largely unknown.

ATP-related cellular and subcellular phenomena include cytoskeletal dynamics^10^ and cellular morphological changes^11^,^12^,^13^. In chick ciliary neurons, ATP depletion suppresses actin turn-over and long-term ATP depletion causes changes in cellular shape^10^. Hippocampal neurons lacking cytoplasmic polyadenylation element binding protein 1 (CPEB1) have brain-specific dysfunctional mitochondria and reduced ATP levels, which result in defective dendrite morphogenesis^11^. Also, in neuronal spines, neuronal activity increases ATP consumption. Synaptic vesicle recycling presents a large ATP burden, which may be because of dynamin that mediates membrane fission^12^. These previous reports indicate that variation in ATP levels is related to cellular morphological changes and cytoskeletal dynamics.

To demonstrate the presence of a direct relationship under physiological conditions, precise and simultaneous observation of ATP levels and either cellular morphology or cytoskeletal dynamics is necessary. This has been difficult because conventional ATP quantification methods do not allow for high-resolution observation^14^. Although the technical development of the novel genetic ATP sensor ATeam enabled such observations^14^, finding the relationships is still not easy, because, in general, fluctuation in biological signals without extensive stimulation is subtle and occurs over a narrow range. Despite this technical challenge, we recently successfully investigated the relationship between the motility of the growth cone and the crosstalk of second messengers through a combination of simultaneous imaging with spatiotemporal image processing analysis^15^.

In this study, we combined simultaneous imaging with detailed analysis to reveal the relationships between cytoskeletal dynamics, morphological change, and ATP level change. We conducted several kinds of simultaneous imaging using ATeam, an indicator for microtubule dynamics that used fluorescent-labeled EB3 (end-binding protein 3)^16^,^17^,^18^, fluorescent-labeled actin, and fluorescent dye for the plasma membrane (FM4-64) in HeLa cells. We quantified the spatiotemporal behavior of the cells using original image processing software, and revealed that cytoskeletal dynamics at the cell edge are related to cellular morphology and intracellular ATP levels, and that actin and microtubules influence them in different ways.

## Results

### Inhibition of cytoskeletal dynamics increases local ATP

**Figure 1:**
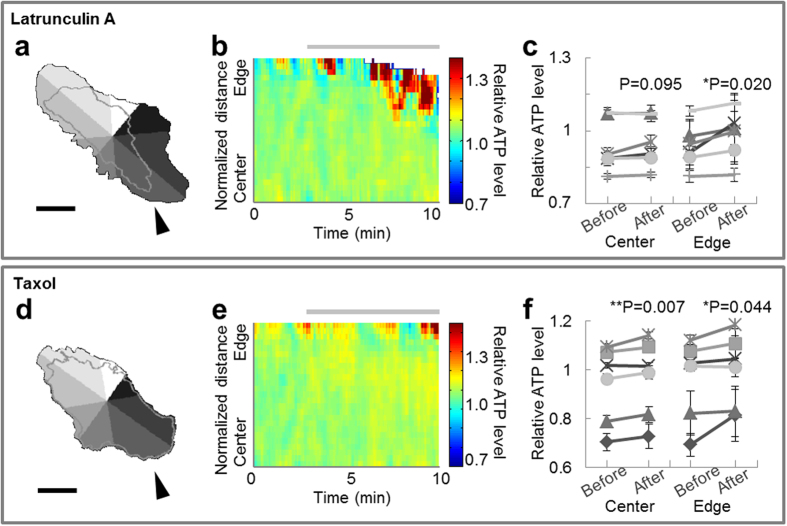
Inhibition of cytoskeletal dynamics induces an increase in local ATP. (**a,d**) Typical images of the cellular morphology at the beginning of the observation. The gray-shaded regions represent 8 automatically divided compartments, while the gray line depicts the cellular morphology at the end of the observation. Scale bar represents 30 μm. (**b,e**) Typical image of the spatiotemporal behavior of intracellular relative ATP levels in the compartment indicated by the arrowhead in the left figure. The horizontal axis indicates time, the vertical axis indicates position, and pseudo color indicates relative ATP level. Each color lookup table is linear and covers the full range of the data. The gray bar indicates the duration of inhibition. (**c,f**) Comparisons of relative ATP level before and after inhibition at the central and edge parts. When cells were treated with Latrunculin A, relative ATP level increased only at the edge (*n* = 6). On the other hand, Taxol-treated cells showed an increase in relative ATP level at both the central and the edge parts (*n* = 6). Error bar represents standard deviation (s.d.).

Our goal was to reveal the relationships between change in intracellular ATP levels, cytoskeletal dynamics, and morphological change in HeLa cells under physiological conditions. To verify whether these relationships exist, we first examined if the inhibition of cytoskeletal dynamics affect intracellular ATP levels. HeLa cells expressing ATeam were imaged under physiological conditions for 10 min, and cytoskeletal dynamics were modulated by 100 nM Latrunculin A or 200 nM Taxol at 3 min. Latrunculin A binds with 1:1 stoichiometry to monometric actin^19^, sequesters monomers, and prevents their reassembly^20^. Latrunculin A-treated cells are known to lose their focal adhesions and retract^21^. Taxol specifically binds to and stabilizes microtubules^22^. Application of Taxol completely abolishes the binding of microtubule-associated proteins to the ends of growing microtubules^17^, therefore disrupting microtubule dynamics^18^. As expected, Latrunculin A caused retraction in 8/8 cells (Fig. 1a). 6/7 Taxol-treated cells also showed morphological change (Fig. 1d). Because the degree of retraction differed by location, we separated each cell into 8 compartments (Fig. 1a,d), and quantified spatiotemporal ATP levels and cellular morphology within each compartment (Fig. 1b,e). Statistical analysis revealed that cells treated with Latrunculin A showed ATP levels that were increased only at the edge part, while Taxol-treated cells exhibited increased ATP levels at both the central and the edge parts (Fig. 1c,f). On the other hand, 10 mM 2-DG (in 3/3 cells) and 1 μM FCCP (carbonyl cyanide-p-trifluoromethoxyphenylhydrazone, in 3/3 cells), which suppress ATP production, lowered relative ATP levels throughout the cell without any place dependency (Supplementary Fig. 1a–f). FCCP is a protonophore, which is known to cause calcium leak from mitochondria, so the leaked calcium might have influences on the cytoskeleton or ATP levels. We also confirmed that FCCP increased calcium levels (Supplementary Fig. 1g), although neither FCCP nor ionomycin, which causes a larger calcium leak than FCCP, induced significant changes in morphology in the cellular areas examined (Supplementary Fig. 1g,h).

From the above, the results suggest cytoskeletal dynamics are related to change in ATP levels, especially at the cell edge. Moreover, the influence upon cellular morphology and ATP levels differs depending on whether actin or microtubule dynamics are involved.

### Cell edge has two marginal structures

**Figure 2:**
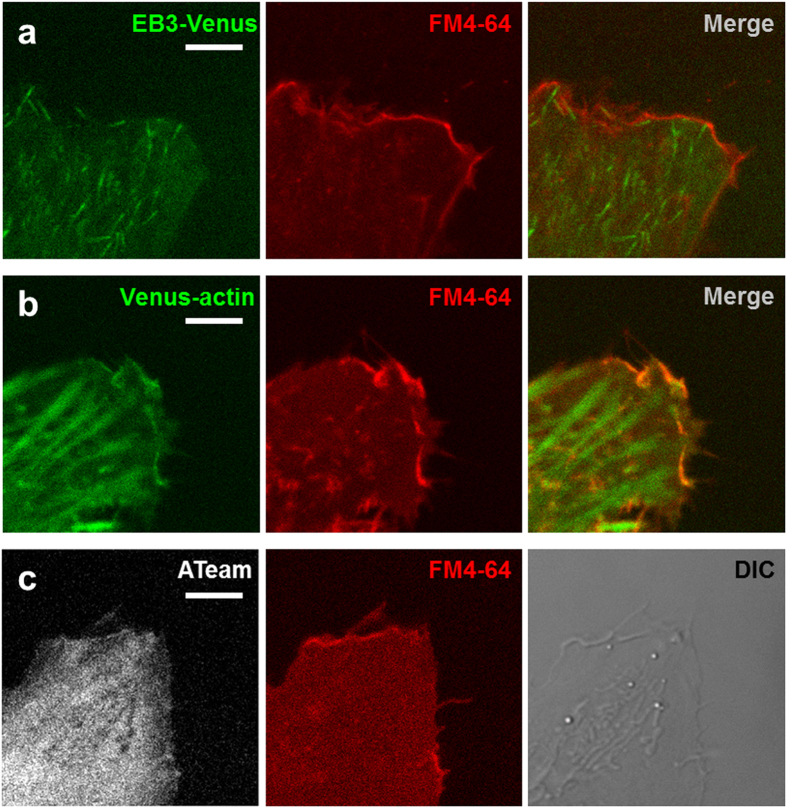
Structure at the cell edge: the lamella and the peripheral structure are distinguished. (**a**) Typical EB3-Venus (left), FM4-64 (middle), and merged (right) images at the edge of a HeLa cell. EB3 probe diffused throughout the cytosol. (**b**) Typical Venus-actin (left), FM4-64 (middle), and merged (right) images at the edge of a HeLa cell. The peripheral structure of the cell edge is constructed of actin filaments. (**c**) Typical images of the (mseCFP + Venus) fluoresce of ATeam (left), FM4-64 (middle), and DIC (right) at the edge of a HeLa cell. The peripheral structure at the cell edge was detectable from the (mseCFP+Venus) signal. Scale bar represents 5 μm.

Cytoskeletal-dynamics-related changes in morphology and intracellular ATP levels were remarkable at the cell edge. We therefore focused the next stages of our research on the cell edge. We considered it necessary to visualize actin and microtubule separately because their influence upon ATP levels and cell morphology were different. We first observed HeLa cells expressing EB3-Venus. EB3 is a protein which binds to the plus end of a microtubule during elongation growth, and fluorescent-labeled EB3 acts as an indicator for microtubule dynamics^16^,^17^,^18^. EB3-Venus was found not only at the plus ends of microtubules, but also diffused throughout the cytosol (Fig. 2a). However, the fluorescent dye for the lipid bilayer, FM4-64, revealed that there is also the peripheral structure consisted of actin, which was not visualized by EB3-Venus (Fig. 2a,b). The region EB3-Venus diffused into is called the ‘lamella’ and the peripheral structures outside the lamella are called ‘filopodia’ or ‘lamellipodia’^23^,^24^. Up to here, we have conceptually grouped these regions by the term ‘edge’, but we will now begin to consider them separately, calling them ‘lamella’ and ‘peripheral structure’, respectively.

### Cell shape change by microtubule accompanying ATP increase

**Figure 3:**
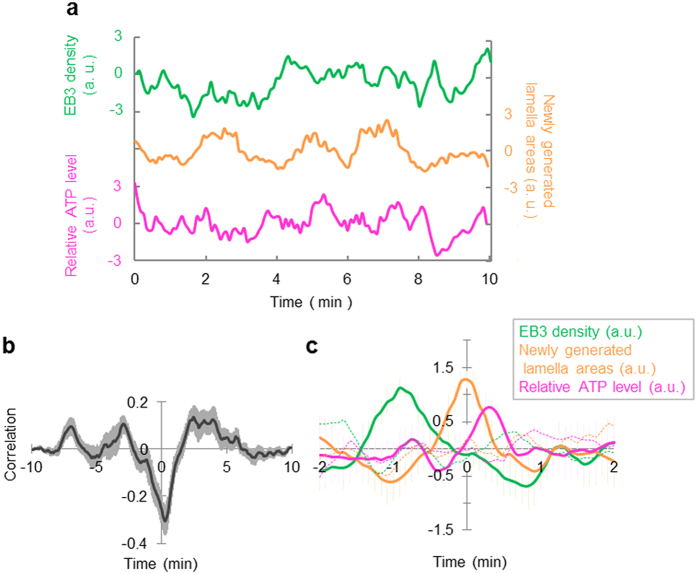
Microtubule dynamics cause a change in cell shape, which is accompanied by an increase in the ATP level at the cell edge. (**a**) Typical time courses of EB3 density (green), the number of newly generated lamella areas (orange), and the relative ATP level (pink) within an ROI. (**b**) Cross correlation function between newly generated lamella areas vs. EB3 density. Positive correlations were observed at −7 min, −2.5 min, and 3 min (12 ROIs from 6 cells). (**c**) An average of waveforms in the duration between before and after the timing of a peak in newly generated lamella areas (solid line, 9 events from 6 cells). EB3 density increased about 1 min before the peak, and this increase in the lamella area accompanied an increase in the relative ATP level. No apparent peak was observed in the average of waveforms from randomly shuffled sequences (broken lines). Error bar represents standard error of mean (s.e.m.).

We explored whether these two layers were related through simultaneous observation of EB3-labeled microtubules and the peripheral structure visualized by FM4-64. These observations revealed that the peripheral structure showed high motility where the dynamics of EB3-labeled microtubule were active (Supplementary Video 1). This peripheral movement was suppressed by Taxol-induced inhibition of microtubule dynamics. As mentioned in many previous reports, disturbance of microtubule dynamics negatively affects actin dynamics and cellular morphology, which could be rescued by actin stabilization^25^,^26^, suggesting that microtubule dynamics are upstream of actin dynamics. Indeed, we found no obvious effects on microtubule dynamics in Latrunculin-A treated cells (Supplementary Fig. 2). So, we focused on microtubule dynamics first, examining the relationships between microtubule dynamics and change in cellular morphology or ATP levels. In EB3-mCherry expressing cells under physiological conditions, microtubules rushed into and touched the lamella boundary, changing the lamella shape (Supplementary Fig. 3 and Supplementary Video 2). Next, we observed HeLa cells co-expressing EB3-mCherry and ATeam to explore how these microtubule dynamics affect intracellular ATP levels. We chose the region of interest (ROI) at the cell edge, and acquired time-series data of EB3 density, the number of newly generated lamella areas, and relative ATP levels (Fig. 3a). We constructed a cross correlation function between newly generated lamella areas and EB3 density (Fig. 3b). Since a negative peak at 0 min is a result of parameter properties (as EB3 density is defined as the value of (EB3 positive pixel)/(cell area positive pixel), it decreases when the newly generated lamella area increases), we focused only on the positive peaks. There were three positive peaks at −7 min, −2.5 min and 3 min, suggesting that cross correlation analysis would be required within 5 min. Then, we averaged the waveforms in the duration between before and after two minutes the timing of a peak in newly generated lamella areas. This analysis showed that EB3 density increases about 1 min before the peak in newly generated lamella areas and that this area growth accompanies an increase in the relative ATP level (Fig. 3c, solid line). This increase was not obvious in the calculations using randomly shuffled datasets (Fig. 3c, broken line) nor signals from the ATP-insensitive C-Y FRET indicator (Supplementary Fig. 4). Moreover, inhibition of microtubule dynamics by Taxol reduced the number of EB3 particles and EB3 contacts with the lamella boundary, therefore suppressing cellular morphological change that was obvious under physiological conditions (Supplementary Fig. 2 and Supplementary Video 3). From the above findings, microtubule rush appear to increase the lamella area at the cell edge and this increase involves a rise in ATP levels.

### Cytoskeletons participate in change in morphology and ATP

**Figure 4:**
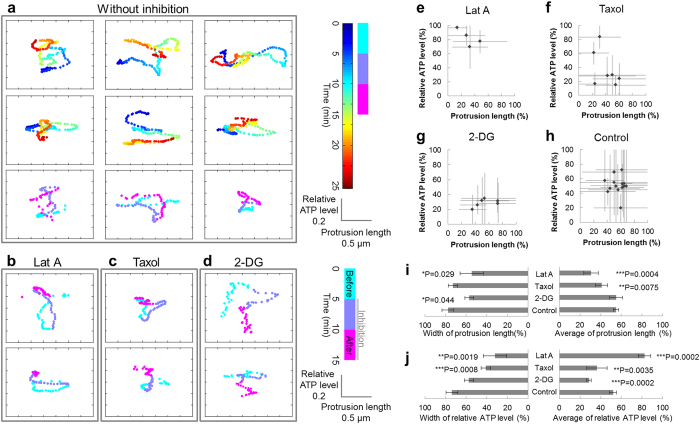
Both actin and microtubule dynamics affect cellular ATP levels at the cell edge. (**a**) Typical tracks of protrusion length and ATP levels at the edges of HeLa cells under physiological conditions (9 examples from 5 cells). The horizontal axis indicates protrusion length, the vertical axis indicates relative ATP levels, and the pseudo color indicates time. (**b–d**) Typical tracks of HeLa cells treated with (**b**) Latrunculin A (left, 2 examples from 2 cells), (**c**) Taxol (middle, 2 examples from 2 cells), or (**d**) 2-DG (right, 2 examples from 2 cells). During the last 5 min of observation (pink), the tracks of Latrunculin A-treated cells lost their movement, while a reduced range of variation in relative ATP levels was observed in the tracks of Taxol-treated cells. Cells treated with 2-DG showed decreased motility as well as ATP levels. (**e**–**h**) Ranges of measured values in the last 5 min as percentages of the total timeframe. Markers indicate the mean values, while bars indicate the ranges. (**i,j**) Statistical analysis of (left) width and (right) average values of the ranges of (**i**) protrusion length and (**j**) relative ATP levels (4 ROIs from 2 cells for Latrunculin A, 7 ROIs from 3 cells for Taxol, 6 ROIs from 2 cells for 2-DG, and 15 ROIs from 6 cells for control).

We revealed that microtubule dynamics are not only associated with change in morphology and ATP levels at the lamella, but to motility of peripheral structure consisting of actin. Actin requires ATP for polymerization, so we posited that ATP level changes observed in the lamella that is related to microtubule dynamics could be linked to the morphological change of the peripheral structure. We observed HeLa cells expressing ATeam with high spatial resolution, which enabled us to collect data on not only ATP levels but also the appearance of the peripheral structure (Fig. 2c). The lamella was assumed to correspond to the region 1–2 μm interior from the cell edge (Fig. 2), and exterior to this lamella region was defined as the peripheral structure. We set a small ROI at the cell edge where morphology dynamically changed, then estimated protrusion length of the peripheral region by measuring distance from the tip of the peripheral structure to the lamella. In addition, we calculated relative ATP level near the protrusion within the lamella and then plotted the time change of relative ATP level and protrusion length. The plot continuously varied within the range of ~0.7 relative ATP level and ~3 μm of protrusion length over 25 min (Fig. 4a,h), visualizing very intricate tracks. To investigate the origin of these complicated tracks, we applied Latrunculin A, Taxol, or 2-DG at 5 min and conducted the imaging for 15 min. Since the inhibition by Latrunculin A, Taxol and 2-DG were ineffective immediately after their application, and the time lags differed among cells, we analyzed the effect of the inhibitors on protrusion length and relative ATP levels in the last 5 min (pink-colored time period in Fig. 4a–d). We calculated the range of relative ATP levels and the protrusion length within the last 5 min as percentages of the total 15 min (Fig. 4e–j). In Latrunculin A-treated cells, the dynamic range of track distance was extremely narrow in the last 5 min. The tracks also converged at the point of a short protrusion length and a high relative ATP level (Fig. 4b,e,i,j). Time-lapse fluorescent imaging also showed that Latrunculin A treatment diminished the peripheral structural area (Supplementary Video 4). Therefore, Latrunculin A reduces the area of the actin-based structure, which could increase ATP levels. The dynamic range of relative ATP levels and the protrusion length of the tracks in the last 5 min decreased when cells were treated with Taxol (Fig. 4c,f,i,j). Time-lapse fluorescent imaging demonstrated that not only the peripheral structure but also the lamella retracted, which is different from the morphological change caused by Latrunculin A (Supplementary Video 5). These observations suggest that Taxol treatment deprived cells of the changes in morphology and the ATP levels observed within the lamella, which resulted in a decrease in protrusion length. On the other hand, in cells treated with 2-DG, tracks in the last 5 min showed a drastic decrease in relative ATP levels as well as scanty cell edge motility (Fig. 4d,g,i,j and Supplementary Video 6). This suggests that ATP would be required for morphological changes. Taken together, these findings indicate that morphological change in the peripheral structure is associated with ATP level change, and while actin dynamics play a great role, microtubule dynamics are also related. We reasoned that these two kinds of influences upon ATP levels and cellular morphology could explain the complexity of the tracks. The tracks showed both right-handed and left-handed rotations and shifted between both rotations sequentially, indicating that ATP level is not always preferentially controlled over the protrusion length, nor *vice versa*. It could be reasonable to consider the complexity of the tracks is a consequence of the complex mixture of these two effects on ATP levels and protrusion length. In summary, at the cell edge, morphological change in peripheral structure and ATP levels are affected primarily by actin dynamics, but also, weakly, by microtubule dynamics.

## Discussion

We conducted various simultaneous imaging and spatiotemporal analyses of intracellular ATP levels, cytoskeletal dynamics, and cellular morphological change, and revealed striking correlations among them.

Actin dynamics are reportedly related to ATP levels^10^ and we confirmed this assertion through experiments with Latrunculin A (Fig. 1a–c and Fig. 4b,e,i,j). Our results indicated that intracellular ATP level at the cell edge is influenced by not only actin dynamics but also microtubule dynamics. Although the effect of microtubule dynamics seemed less distinct than that of actin (Fig. 4b,c,e,f,i,j), a combination of simultaneous imaging and image processing analyses enabled us to demonstrate its presence.

Cultured hippocampal neurons derived from CPEB1 knock out (KO) mice showed a decrease in ATP production and fewer dendrite branches^11^. When these CPEB1 KO neurons were cultured in medium containing phosphocreatine for 4 days *in vitro*, ATP levels recovered to nearly wild type levels, as did dendritic branches. Although these results suggest ATP is important for neurite outgrowth and morphogenesis, the relevance between them were not asserted. This is because intracellular ATP levels and cell morphology were observed in different cells: that is, not coincidently. Our simultaneous imaging conducted here succeeded in directly showing that cytoskeletal dynamics induce changes in intracellular ATP levels and cellular morphology.

Several cellular mechanisms can produce spatiotemporal heterogeneity in ATP distribution. The first mechanism is the localization and the level of production of ATP in mitochondria. In islet 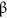 cells, glucose treatment produces the microdomain of ATP beneath the plasma membrane, which is guessed because peripherally located mitochondria are regulated differently from mitochondria in the rest of the cytosol^27^. Mitochondria were confirmed at the lamella in our experiments (Supplementary Video 4–6); hence, these peripheral mitochondria would produce spatiotemporal heterogeneity of ATP in HeLa cells. Another possible mechanism is the consumption of ATP by ATPase or cytoskeleton during morphological changes. As shown in Fig. 1b,c and Fig. 4b,e,i,j, ATP is consumed during actin polymerization. Additionally, since cellular morphological change accompanies the morphological change of cellular membrane, ATPase function during this process should be considered as well. For example, dynamin, known to be mainly active in membrane fission, has been discovered in various dynamic membrane structures, such as lamellipodia at the leading edge of moving cells^28^,^29^. Besides dynamin, soluble N-ethylmaleimide-sensitive factor attached protein receptor (SNARE)-related proteins such as vesicle-associated membrane proteins (VAMPs) are also associated with cellular morphology in epithelial cells^30^. Being active events at the cell edge, actin dynamics and membrane morphological change could also result in ATP heterogeneity.

In summary, we explored the spatiotemporal behavior of HeLa cells in terms of ATP level change, cellular morphological change, and cytoskeletal dynamics under physiological conditions. Simultaneous imaging and detailed image processing revealed that, at the cell edge, both actin dynamics and microtubule dynamics are inextricably tied to changes in intracellular ATP levels and cellular morphology.

## Methods

### Materials

FM4-64 was purchased from Invitrogen, Latrunculin A was from TOCRIS, and Taxol and 2-DG was from Sigma-Aldrich.

### Plasmid construction

The ATeam 1.03 plasmid was kindly provided by Prof. Imamura (Kyoto University, Kyoto, Japan). EB3-mCherry and EB3-Venus were constructed in our laboratory. EB3 was derived from Human cDNA library and amplified using PCR. PCR products were digested with BamHI and NotI, and cloned into a pcDNA3.1(+) vector inserted into mCherry or Venus at NotI and XhoI sites.

### Cell culture

HeLa cells were cultured in Dulbecco’s modified Eagle’s medium (DMEM, Invitrogen) supplemented with 10% FBS (NICHIREI BIOSCIENCE INC) and 1% penicillin/streptomycin (Nacalai Tesque). Cells were maintained at 37 °C in a humidified atmosphere of 5% CO_2_. One to two days before transfection, cells were plated at 1~2 × 10^4^ cells/cm^3^ onto a glass-based dish (IWAKI).

### Transient transfection

Cells were transfected using lipofectamine LTX (Invitrogen), provided that construct concentrations were 1 μg/dish for ATeam, 0.5 μg/dish for EB3 probes, and 2 μg/dish for Venus-actin. After the transfection, medium was changed to Phenol red-free culture medium. Imaging was conducted 12–36 h after transfection.

### Fluorescence microscopy

All fluorescent imaging experiments were performed using a confocal laser-scanning microscope (FV1000 IX81, OLYMPUS) with a ×100 oil immersion objective lens. Furthermore, ×5 optical zoom was used to focus on the cell edge. FM4-64 (200 μg/mL) was applied 50–100 μL/dish before acquisition of the images.

In whole cell imaging, ATeam was excited by a diode laser (440 nm) through a dichroic mirror 405–440/515. The emitted fluorescence was separated by a 510 nm dichroic mirror, and signals from mseCFP and Venus were observed at 460–500 nm and 515–545 nm, respectively.

For simultaneous imaging of ATeam and EB3-mCherry, ATeam and mCherry were excited by the diode laser (440 nm) and a helium-neon laser (559 nm), respectively, through a beam splitter 20/80. The fluorescence was separated by dichroic mirrors (510 nm and 560 nm), and signals from mseCFP, Venus, and mCherry were observed at 475–500 nm, 510–530 nm, and 575–675 nm, respectively.

In simultaneous imaging of EB3-Venus or actin-Venus and FM4-64, Venus and FM4-64 were simultaneously excited by an argon (Ar) laser (515 nm) through a beam splitter 20/80. The emitted fluorescence was separated by a 560 nm dichroic mirror and signals from Venus and FM4-64 were observed at 535–545 nm and 620–720 nm, respectively. Images were acquired with a resolution of 640 × 640 pixels (actual size: 0.039 μm/pixel).

For simultaneous imaging of ATeam and FM4-64, these probes were excited by the diode laser (440 nm) and the Ar laser (515 nm), respectively, through the beam splitter 20/80. The emitted fluorescence was separated by dichroic mirrors (510 nm and 560 nm) and signals from mseCFP, Venus, and FM4-64 were observed at 460–500 nm, 535–560 nm, and 610–710 nm, respectively. Images were acquired with a resolution of 640 × 640 pixels (actual size: 0.039 μm/pixel).

In whole-cell ATeam imaging and the simultaneous imaging at the cell edge, images were acquired with a resolution of 320 × 320 pixels (actual size: 0.397 μm/pixel) every 5 sec. In high-resolution ATeam imaging at the cell edge, images were acquired with a resolution of 640 × 640 pixels (actual size: 0.039 μm/pixel) every 10 sec. During imaging, cells were maintained at 37 °C using a stage heater (TOKAI HIT).

### Image processing and analysis

Images were analyzed with original software written and developed in Matlab (MathWorks). All images were median-filtered before the following steps:

*Detection of cellular morphology:* Median-filtered images were binarized and labeled. The area with the largest number of pixels was determined to be the cell (pixels to be included in the cell are called ‘cell pixels’).

*Relative ATP level calculation:* Relative ATP level was determined by calculating pixel-by-pixel Venus/mseCFP value (called ‘FRET value’ below). Outlier FRET values were removed.

*EB3 particle detection:* A kernel large enough to contain one EB3 particle was prepared. The central pixel of the kernel was labeled 1 if the value of the pixel was more than (*μ* + *σ*), otherwise 0 (*μ* and *σ* refer to the average and the standard deviation of the intensities of the pixels within the kernel, respectively). Binarized images were labeled and the area with a sufficiently large size was designated as an EB3 particle. (Pixels included in an EB3 particle are called ‘EB3 pixels’ below.)

*Unmixing processing:* In analysis of simultaneous imaging, unmixing processing was conducted before calculating relative ATP levels or detecting EB3 particles. HeLa cells expressing mseCFP, Venus, or mCherry were prepared beforehand and any fluorescence leaked from one detection channel to another was measured for each permutation of channels under observation conditions. Then, a 3 × 3 matrix was derived by arranging the leakage values of each permutation. Intrinsic fluorescence was calculated by multiplying observed fluorescence by the inverse matrix of the derived matrix (Supplementary Fig. 5).

*Whole cell analysis:* The long axis, the short axis, and two straight lines 45 degrees to the long axis were set for cell morphology observed at *t* = 0, by which the cell was sectioned into 8 compartments.

*Analysis of the relationships among EB3 particle movements, cellular morphological changes, and relative ATP levels:* Analysis was conducted within small, manually set ROIs at the cell edge where there are dynamic changes in morphology. EB3 density was calculated by dividing the number of EB3 pixels by the number of cell pixels. ATP level was the average of the FRET values of cell pixels. Cellular morphology at *t* = *t* was subtracted from that at *t* = *t* + 1, and the remaining pixels were considered as a newly generated cell area. To analyze how relative ATP levels behave during microtubule-related increase in the lamella area, we drew Fig. 3c through the following steps. First, a time window (length: 4 min) was applied to a peak in the newly generated lamella area. Second, time periods that satisfied all of the following conditions were selected: i) the peak exceeds 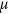
+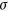 (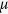 and 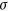 refer to the average and the standard deviation of the value within the time window, respectively); ii) the time window contains only one peak; iii) there is a peak in EB3 density before the peak in the newly generated lamella area; iv) the peak occurs 2 min after the start and 2 min before the end of the observation. The selected waveforms were normalized and then averaged. Finally, Fig. 3c was generated by applying 5 frames of moving average.

*Evaluation of the relationship between protrusion length and relative ATP levels:* Raw images were first processed with median filter and background subtraction. Next, we detected cell form from averaged images of mseCFP and Venus. The lamella area was then determined by selecting a region where a square kernel (radius: 1.75 μm) could be completely contained, and the exterior region was defined as the peripheral structure (Supplementary Fig. 6c; we selected 1.75 μm because the lamella appeared to be 2 μm inside of the boundary of the peripheral structure in our observation; although the peripheral structure estimated by this method appears larger than the real image (Fig. 2), we considered that it would have no significant impact because the differences of protrusion length are presented in Fig. 4). We then set a small ROI (of the same size as was used in the analysis in Fig. 3) at the cell edge where there are dynamic changes in morphology. Protrusion length was estimated by averaging the length of the peripheral structure. Relative ATP levels were calculated by averaging the FRET value within the area that is 2 μm inside of the boundary of the lamella (Supplementary Fig. 6f; we determined the region to be 2 μm inside of the boundary to avoid striking changes in the region size for calculating FRET values during changes in morphology). Finally, we applied moving average (3 min before and after for physiological conditions, and 1 min before and after for inhibitor-treated conditions) to the protrusion length and relative ATP levels, and plotted the values using pseudo color.

### Statistical Analysis

To compare the relative ATP levels from before and after inhibition of cytoskeletal dynamics at the center and edge parts of the cell, we averaged the values of the 3^rd^–23^rd^ pictures (15 seconds–115 seconds from the onset of observation) and that of the 97^th^–117^th^ pictures (365 seconds–465 seconds from the inhibition). In Fig. 1, ‘center’ means the area 10–20% from the center of the cell and ‘edge’ means the area 10–20% from the cell edge. Data were evaluated by Student’s t-test.

## Additional Information

**How to cite this article**: Suzuki, R. *et al.* Spatiotemporal quantification of subcellular ATP levels in a single HeLa cell during changes in morphology. *Sci. Rep.* **5**, 16874; doi: 10.1038/srep16874 (2015).

## Supplementary Material

Supplementary Information

Supplementary Video 1

Supplementary Video 2

Supplementary Video 3

Supplementary Video 4

Supplementary Video 5

Supplementary Video 6
