# Supplementary material for: Spatiotemporal quantification of subcellular ATP levels in a single HeLa cell during changes in morphology
Source: Sci Rep. 2015 Nov 17;5:16874. doi: 10.1038/srep16874 (PMC4647183; doi:10.1038/srep16874)
Supplement: Supplementary Information [file srep16874-s1.pdf]

Supplementary Information

Spatiotemporal quantification of subcellular ATP levels in a single HeLa cell during changes in morphology

Rika Suzuki, Kohji Hotta, and Kotaro Oka*

Supplementary Figure 1

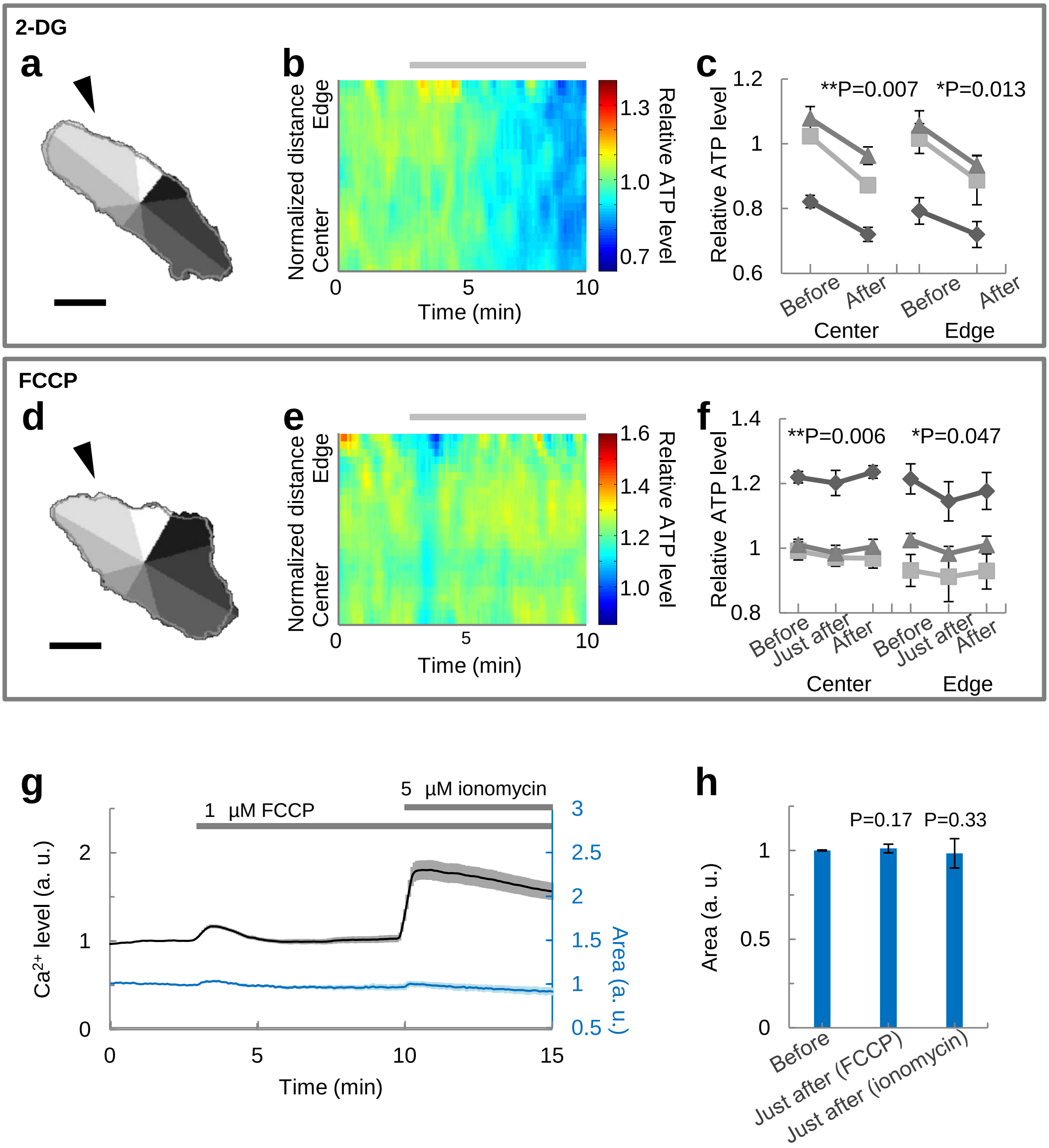

Supplementary Figure 1 Inhibition of ATP synthesis reduced ATP levels throughout the cell. (a,d) Typical images of the cellular morphology at the beginning of the observation. The gray-shaded regions represent 8 automatically divided compartments, while the gray line depicts the cellular morphology at the end of the observation. Scale bar represents 30 μm. (b,e) Typical images of the spatiotemporal behavior of intracellular relative ATP levels in the compartment indicated by the arrowhead in the left figure. The horizontal axis indicates time, the vertical axis indicates position, and the pseudo color indicates the ATP level. Each color lookup table is linear and covers the full range of the data. The gray bar indicates the duration of inhibition. (c,f) Comparisons of ATP levels before and after the inhibition at the central and edge parts. When cells were treated with glycolysis inhibitor 2-DG, ATP levels decreased throughout the cell (n=3). Mitochondria uncoupler carbonyl cyanide-p-trifluoromethoxyphenylhydrazone (FCCP)-treated cells showed reduced ATP levels just after inhibition but then recovered; the decrease occurred throughout the cell (n=3). Error bar represents s.d. (g) Changes in cytosolic calcium levels (black, left axis) and cell area (blue, right axis). Cells were loaded with Fluo-4 AM by incubation with 10 μM Fluo-4 AM with 0.02 w/v% F-127 for 30 min followed by a 15 min de-esterification period. 1 μM FCCP was applied at 3 min and 5 μM ionomycin was added at 10 min during the 15 min imaging. FCCP caused a slight transient increase in calcium levels (30 cells from 5 dishes). Error bar represents s.e.m. (h) Statistical evaluation of changes in cell area. Application of neither FCCP nor ionomycin induced a significant change in cell area (30 cells from 5 dishes). Error bar represents s.e.m.

Supplementary Figure 2

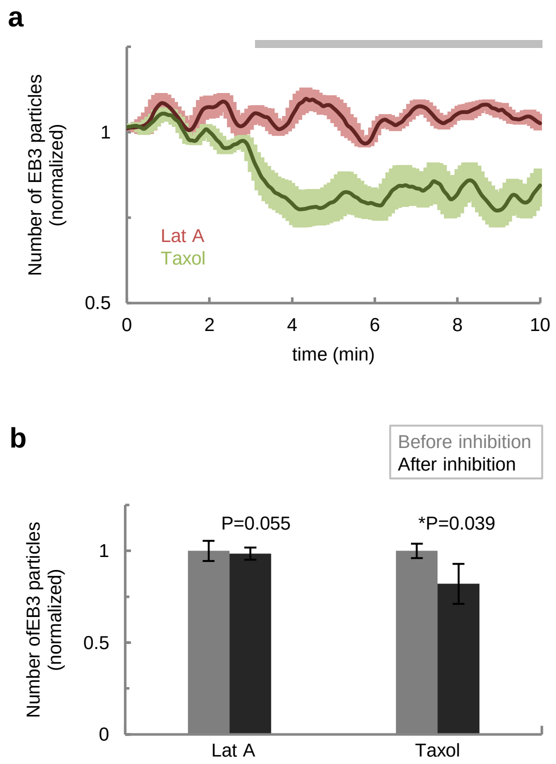

Supplementary Figure 2 Taxol reduced the number of EB3 particles. (a) Averaged time-course of the number of EB3 particles at the cell edge when cells were treated with Latrunculin A (*n*=5) or Taxol (*n*=5). The gray bar indicates the duration of inhibition. (b) Statistical analysis revealed the number of EB3 particles was reduced by Taxol, but not by Latrunculin A. Error bar represents s.e.m.

Supplementary Figure 3

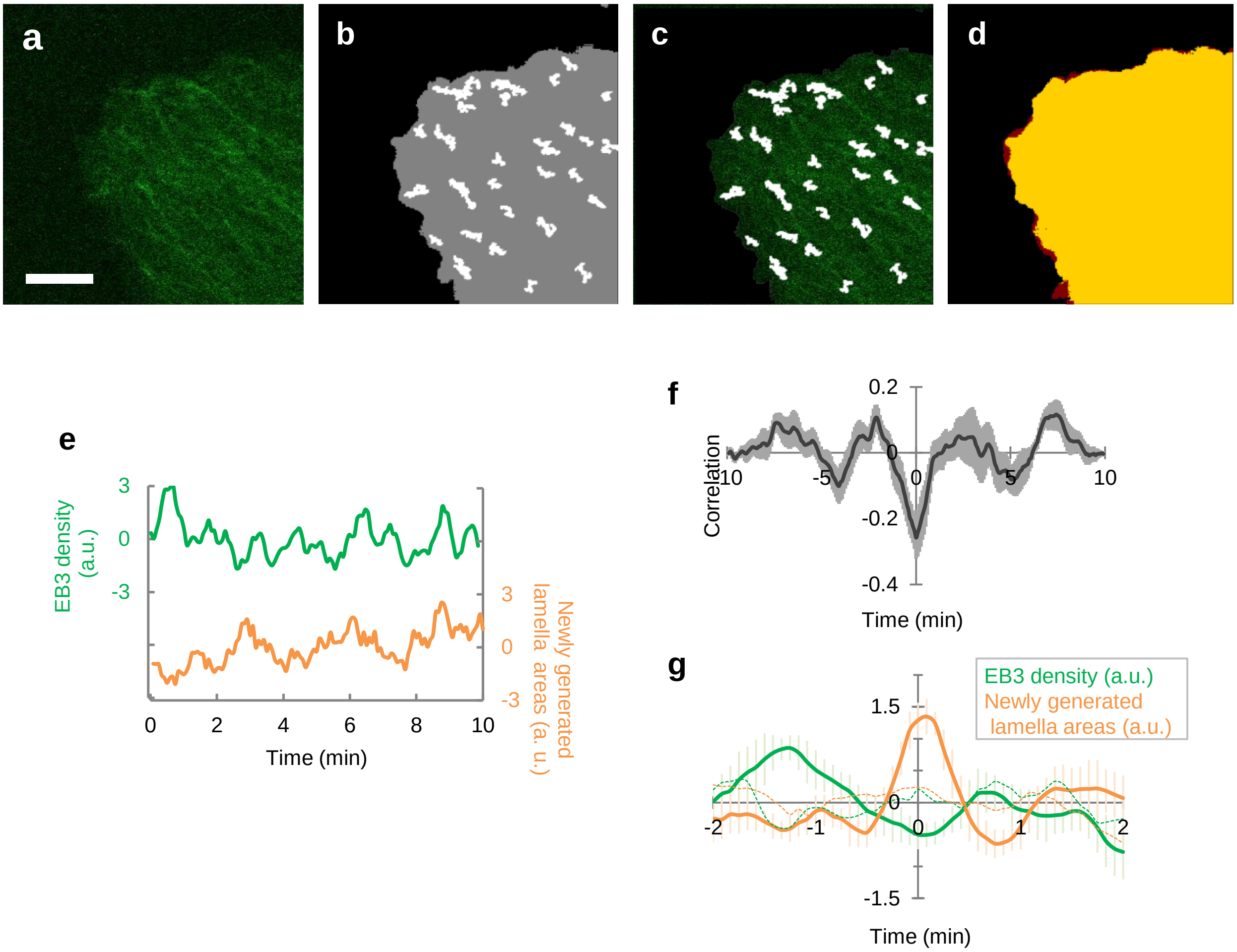

Supplementary Figure 3 Microtubule dynamics cause HeLa cells to change shape. (a) A typical image of the edge of a HeLa cell expressing EB3-mCherry. (b) Detected EB3 particles. Scale bar represents 5 μm. (c) Overlapped image of Supplementary Figure 3a and Supplementary Figure 3b. (d) Time-differential image of cell morphology. Red-colored pixels represent newly generated pixels. (e) Typical time courses of EB3 density (green) and the number of newly generated lamella areas (orange) within an ROI. (f) Cross correlation function between the number of newly generated lamella areas vs. EB3 density. Positive correlation was observed every 5 min (11 ROIs from 8 cells). (g) An average of waveforms in duration between before and after the timing when the number of newly generated lamella areas show peak (8 events from 8 cells). EB3 density increases about 1 to 2 min before the peak. No apparent peak was observed in the average of waveforms from randomly shuffled datasets (broken lines). Error bar represents s.e.m.

Supplementary Figure 4

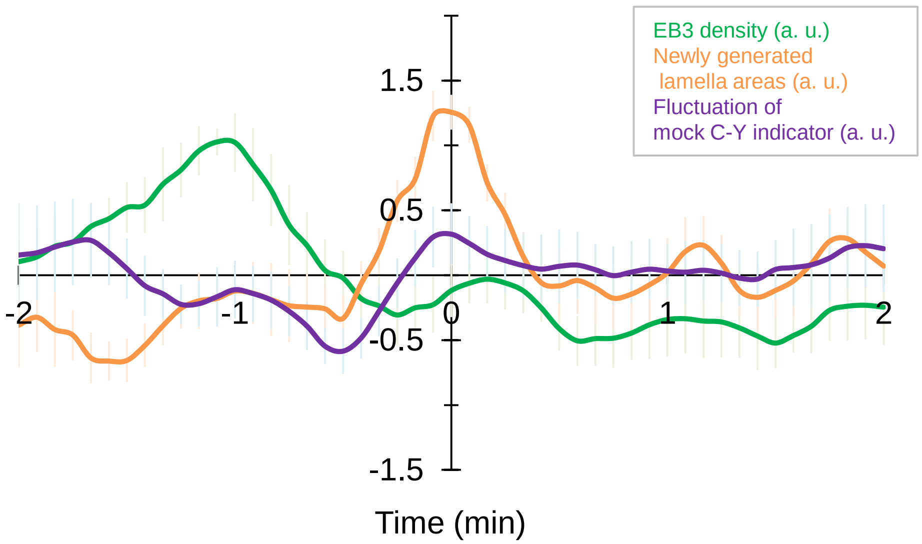

Supplementary Figure 4 No peak was derived from ATP-insensitive FRET sensor. An average of waveforms in the duration between before and after the timing of a peak in newly generated lamella areas (16 events from 10 cells). EB3 density increased about 1 min before the peak. However, the mock Y-C indicator, a construct with CFP and YFP directly connected with a linker, showed any apparent peak unlike ATeam. Error bars represent standard error of mean (s.e.m.).

Supplementary Figure 5

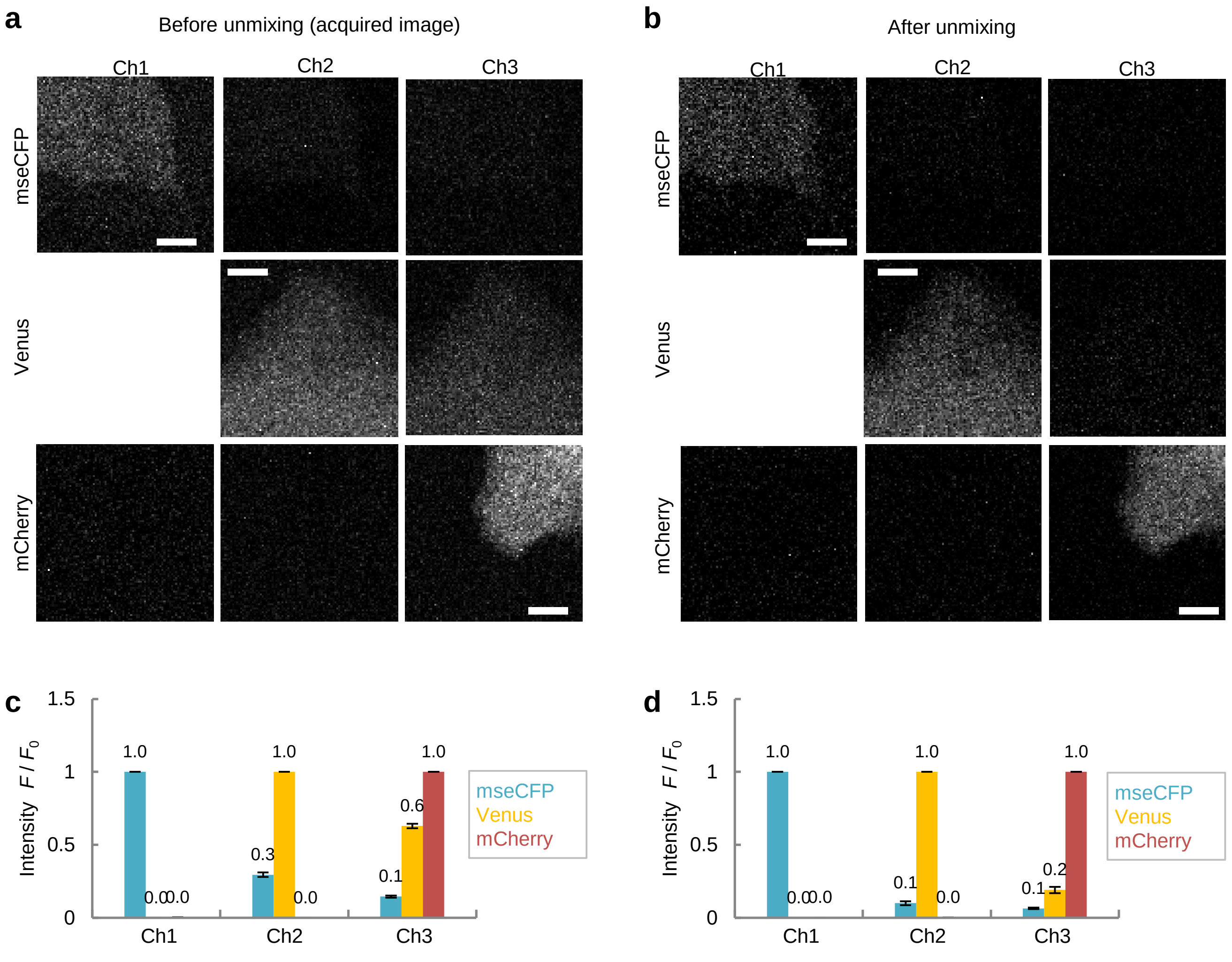

Supplementary Figure 5 Unmixing processing. (a,b) Typical fluorescent images of HeLa cells expressing mseCFP (upper), Venus (middle), or mCherry (lower) acquired at Ch1 (475–500 nm), Ch2 (510–530 nm), and Ch3 (575–675 nm), before (a) and after (b) unmixing processing. Scale bar represents 5 μm. (c,d) The proportions of fluorescence consist each channels. Compared with before unmixing (c), leaked fluorescence was reduced after unmixing (d). Error bar represents s.e.m.

Supplementary Figure 6

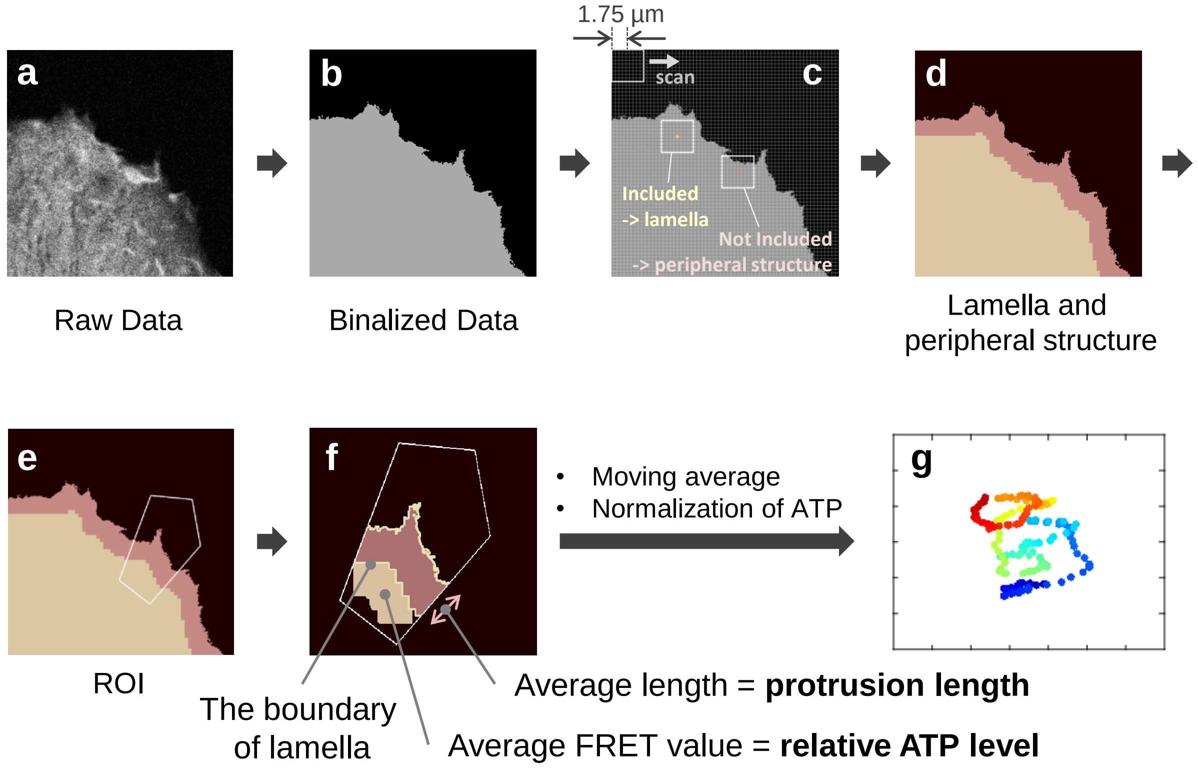

Supplementary Figure 6 Evaluation of the relationship between protrusion length and relative ATP levels. (a) Averaged image of mseCFP and Venus. (b) Detected cell form. (c,d) Lamella area was determined by selecting a region where a square kernel (radius: 1.75 μm) could be completely contained, and exterior region was defined as the peripheral structure. (e) A small ROI was set at the cell edge where there are dynamic changes to morphology. (f) Protrusion length was estimated by averaging the length of the peripheral structure. Relative ATP levels were calculated by averaging the FRET value within the area that is 2 μm inside of the boundary of the lamella. (g) Moving averaged values of protrusion length and relative ATP levels were plotted with pseudo color.

Supplementary Video 1

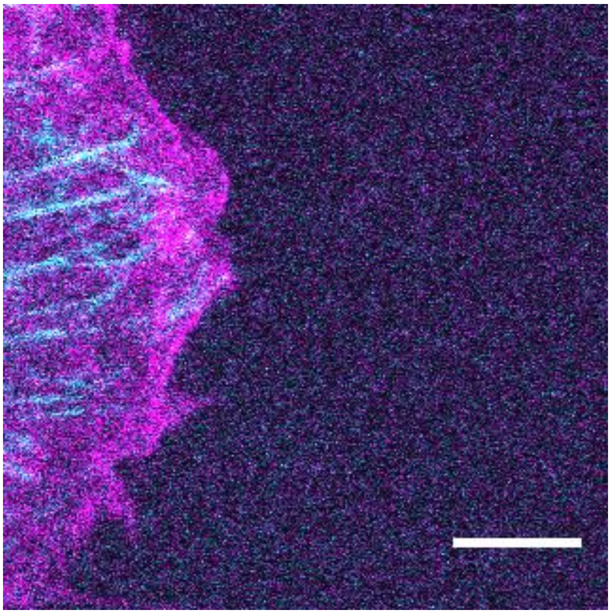

Supplementary Video 1 Peripheral structure shows high motility where microtubule dynamics are active. Typical movie of simultaneous imaging of EB3-Venus (cyan) and FM4-64 (magenta). Peripheral structure visualized by FM4-64 showed high motility where EB3-Venus rushed into the lamella boundary. Scale bar represents 5 μm.

Supplementary Video 2

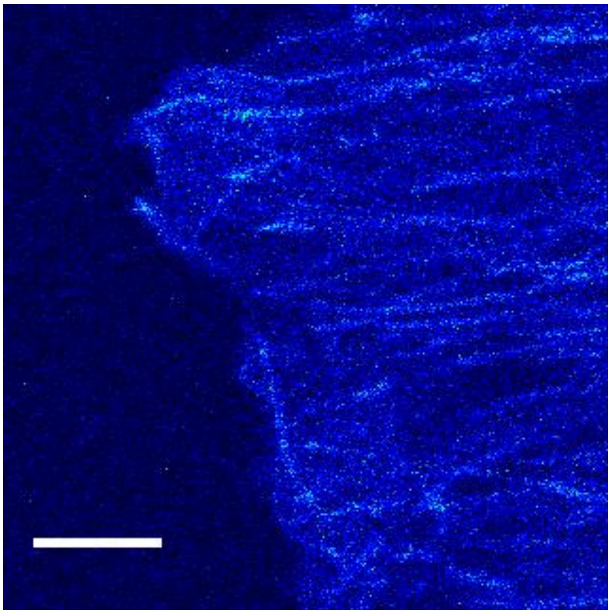

Supplementary Video 2 EB3-labeled microtubules rush into and contact the lamella boundary, causing morphological change. Typical movie of the edge of a HeLa cell expressing EB3-mCherry. Bright spots are EB3 particles and the lamella is visualized by diffused probes. EB3 particles rush into and contact the lamella boundary, resulting in morphological change of the lamella. Scale bar represents 5 μm.

Supplementary Video 3

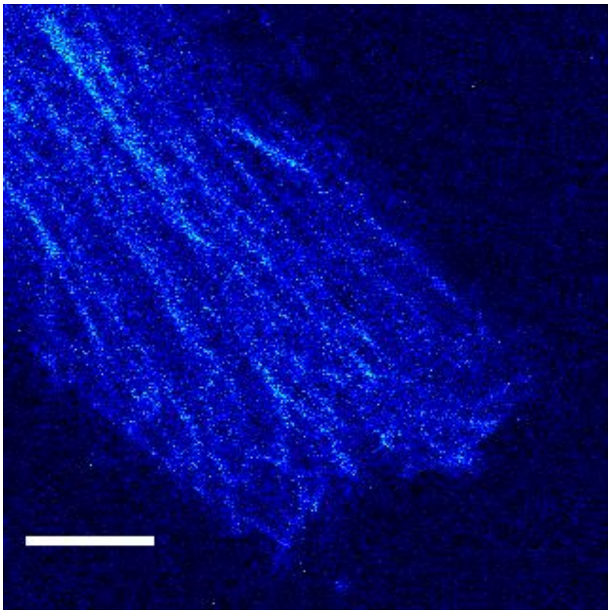

Supplementary Video 3 Microtubule dynamics and morphological change of the lamella are suppressed by Taxol. Typical movie of the edge of a HeLa cell expressing EB3-mCherry. Cells were treated with Taxol at 3 min. Taxol diminished the number and speed of EB3 particles: that is, microtubule dynamics slowed, and morphological change became less active. Scale bar represents 5 μm.

Supplementary Video 4

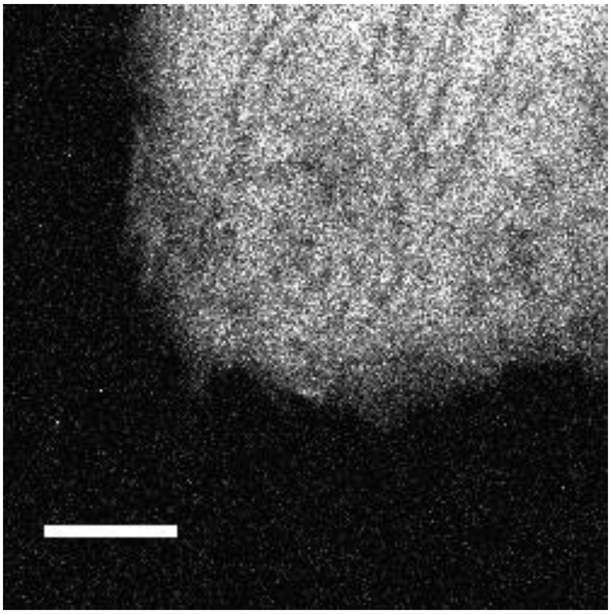

Supplementary Video 4 Latrunculin A reduces the peripheral structure. Typical movie of the edge of a HeLa cell expressing ATeam. Cells were treated with Latrunculin A at 5 min. After inhibition, the motility and area of the peripheral structure decreased. Scale bar represents 5 μm.

Supplementary Video 5

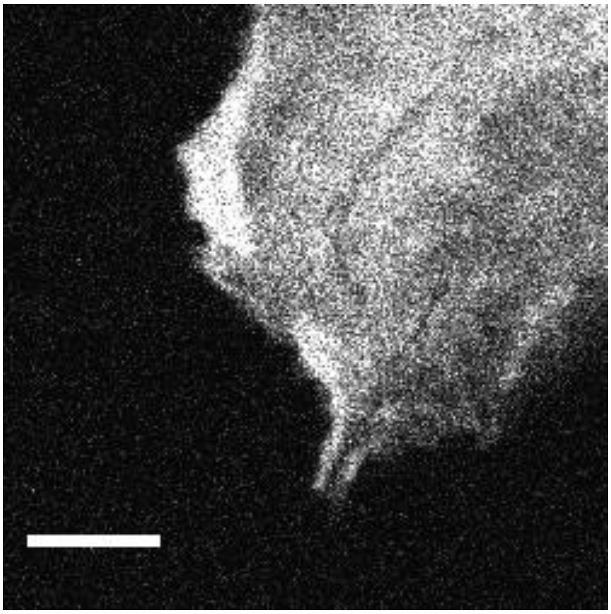

Supplementary Video 5 Taxol affects cellular morphological change in both lamella and peripheral structure. Typical movie of the edge of a HeLa cell expressing ATeam. Cells were treated with Taxol at 5 min. After inhibition, not only the peripheral structure but also the lamella exhibited lower activity. Scale bar represents 5 μm.

Supplementary Video 6

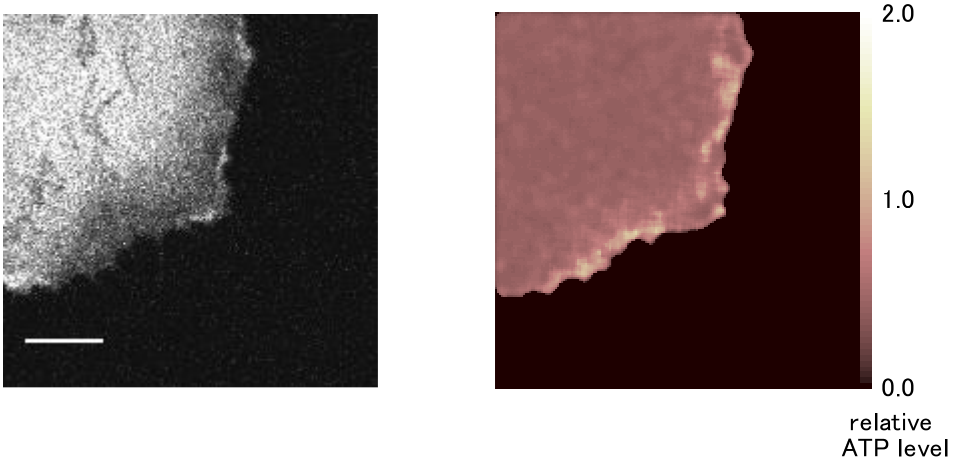

Supplementary Video 6 Lowered ATP level decreased the motility of peripheral structure. Typical movie of morphology (left) and relative ATP level (right) of the edge of a HeLa cell. Color lookup table is linear and covers the full range of the data. Cells were treated with 2-DG at 5 min. After treatment, motility of peripheral structure decreased as ATP level descended. Scale bar represents 5 μm.
